# Photoperiod-Dependent Nutrient Accumulation in Rice Cultivated in Plant Factories: A Comparative Metabolomic Analysis

**DOI:** 10.3390/foods13101544

**Published:** 2024-05-16

**Authors:** Jingyao Yu, Yu Yang, Lanjun Luo, Fang Feng, Sana Saeed, Jie Luo, Chuanying Fang, Junjie Zhou, Kang Li

**Affiliations:** 1 School of Breeding and Multiplication (Sanya Institute of Breeding and Multiplication), Hainan University, Sanya 572025, China; jingyaofish@163.com (J.Y.); y137139636@163.com (Y.Y.); jie.luo@hainanu.edu.cn (J.L.); cyfang@hainanu.edu.cn (C.F.); 2 School of Tropical Agriculture and Forestry, Hainan University, Haikou 570288, China; luolanjun1029@163.com; 3 Wuhan Greenfafa Institute of Novel Genechip R&D Co., Ltd., Wuhan 430070, China; fengfang@greenfafa.com; 4 Department of Plant Breeding & Genetics, University of Sargodha, Sargodha 40100, Pakistan; saeedsana140@gmail.com; 5 School of Life and Health Sciences, Hainan University, Haikou 570288, China

**Keywords:** rice seed, photoperiod, plant factory, vitamin B6, metabolome

## Abstract

Plant factories offer a promising solution to some of the challenges facing traditional agriculture, allowing for year-round rapid production of plant-derived foods. However, the effects of conditions in plant factories on metabolic nutrients remain to be explored. In this study, we used three rice accessions (KongYu131, HuangHuaZhan, and Kam Sweet Rice) as objectives, which were planted in a plant factory with strict photoperiods that are long-day (12 h light/12 h dark) or short-day (8 h light/16 h dark). A total of 438 metabolites were detected in the harvested rice grains. The difference in photoperiod leads to a different accumulation of metabolites in rice grains. Most metabolites accumulated significantly higher levels under the short-day condition than the long-day condition. Differentially accumulated metabolites were enriched in the amino acids and vitamin B6 pathway. Asparagine, pyridoxamine, and pyridoxine are key metabolites that accumulate at higher levels in rice grains harvested from the short-day photoperiod. This study reveals the photoperiod-dependent metabolomic differences in rice cultivated in plant factories, especially the metabolic profiling of taste- and nutrition-related compounds.

## 1. Introduction

Plant factories, also known as vertical or indoor farms, are controlled environments where plants are grown using artificial light, temperature control, and nutrient solutions instead of natural sunlight, soil, and traditional agricultural methods. Diverse photoperiods were used to optimize plant growth [1]. Plants consider changes in photoperiod as seasonal signals, responding to repeated natural stimuli and exhibiting circular growth patterns [2]. According to the response of plant flowering to sunlight length, the photoperiod can be divided into long-day (LD), short-day (SD), and medium-day (MD) plants. The photoperiod drives rhythmic changes in plant behavior, physiology, and metabolism [3]. The most significant response of plants to the photoperiod is the regulation of flowering time. In addition, seed size and stress tolerance are also affected by the photoperiod [2,4,5,6,7]. Therefore, the photoperiod is the most prominent environmental factor that shapes plant structure and determines the transition of growth stages [8,9]. However, we know little about how artificial photoperiods in plant factories affect plant nutritional metabolism.

Light plays a critical role due to its indispensable role in plant growth and development [10]. The presence or absence of light significantly alters the composition of plant metabolites, leading to a profound reprogramming of the plant metabolome [11,12]. The intensity of light is a crucial factor that influences the accumulation of plant metabolites. Intense light exposure promotes the biosynthesis of flavonoids in tea leaves, especially the accumulation of flavonol glycosides and anthocyanins [13]. Conversely, shading, i.e., weak light, diminished the build-up of bitter compounds in tea leaves [14]. In addition, it significantly increased the chlorophyll and amino acid content in tea leaves, leading to a greener color, fresher taste, improved tea flavor, and enhanced quality of matcha [15]. In addition, the duration of light exposure is an additional light factor that impacts the accumulation of metabolites. Long-term exposure to sunlight has been found to enhance significantly the accumulation of anthocyanins in the peel of apples [16,17]. With the extension of light exposure time, the chlorophyll, soluble sugars, ergot amide, and ergometrine contents in the symbiotic seedlings of endophytic fungi in drunken horse grass gradually increased [18]. Moreover, the combination of light intensity and light duration also significantly regulated the accumulation of metabolites. In green alga, *Chlorella ohadii*, short-term high-intensity light exposure stimulated metabolism related to sugars, respiratory intermediates, several primary amino acids, and glycerol. In contrast, continuous high-intensity light treatment caused the accumulation of metabolites related to stress response, photorespiration, and ammonium assimilation [19]. Furthermore, numerous studies have shown that a diversely colored light spectrum have broad regulatory effects on metabolism [20,21]. Supplementing red light significantly triggers melatonin accumulation in tomatoes, improves agronomic traits, and provides additional health advantages [22]. Among the factors influenced by light, an appropriate photoperiod can promote the accumulation of metabolites [23]. The photoperiod controls the content of metabolites in the embryonic callus tissue of Longan. The content and yield of flavonoids were highest under short light treatment (6 h light/18 h dark), while the content and yield of carotenoids were highest under full light treatment (24 h) [24]. The hairy roots of *Hypericum perforatum* L. synthesize five oxanthraquinones, three flavonoid glycosides, and two phenolic acids from scratch under a photoperiod of 16 h/8 h. The production of quinic acid, kaempferol, and seven identified xanthones increased. However, roots under dark cultivation produced more flavane-3-ol [25]. Although advances have been made in studying photoperiod regulation of plant metabolisms, it still needs to be determined how photoperiods affect the metabolome changes of food crops, especially nutritional metabolites, which is extremely important for improving food crop nutrition to ensure food security.

Plant metabolites refer to small molecular compounds that are essential for biological metabolism and required for plant growth and development [26,27]. The simultaneous measure of all metabolites in a particular biological system is metabolomics [28,29]. To discover the metabolic characteristics of organisms more comprehensively, a series of metabolomic techniques, such as liquid chromatography (LC), gas chromatography (GC), and mass spectrometry (MS), have been continuously developed and applied [30,31,32]. Meanwhile, metabolomics is divided into targeted metabolomics and untargeted metabolomics, with a more developed novel integrated method known as widely targeted metabolomics [33]. The widely targeted method of LC-MS is commonly used to separate and detect metabolites in all kinds of samples [34]. It has the advantages of high throughput and high sensitivity, allowing it to more directly and accurately reflect the metabolic state of the organism [35]. In recent years, metabolomics research has been applied to many species, such as maize, rice, tomatoes, kiwifruit, and coconuts [11,36,37,38,39]. These studies provide a comprehensive understanding of the organisms at the individual level, and establish an essential link in explaining the interactions between organisms and the environment [27].

Rice (*Oryza sativa* L.), a typical short-day plant, is one of the most important food crops in the world. The duration of light exposure determines the transition from vegetative to reproductive growth in rice. Thus, we explored the effects of photoperiods on rice’s nutritional metabolome. We cultivated three distinct rice accessions (KY131, HHZ, and KSR) in a plant factory. This study subjected these three varieties to strict short-day (SD) and long-day (LD) treatments. After harvesting, we measured the metabolic profile and found that most metabolites accumulated more under SD. Amino acids and vitamin B6 were essential differentially accumulated metabolites caused by the photoperiod. This study provides the first comprehensive understanding on the influence of the photoperiod on the accumulation of nutrients in rice grains, which is necessary for crop nutrition improvements.

## 2. Materials and Methods

### 2.1. Plant Materials and Growth Conditions

Three rice varieties (KY131, HHZ, KSR) were used in this study: Kongyu131 (KY131) is a japonica accession from the northeast of China; Huanghuazhan (HHZ), an indica rice variety from the south of China; and Kam sweet rice (KSR) is a sticky rice from the Dong nationality in China with high nutritional characteristics [40].

The rice seeds were washed with clean water 3–5 times, immersed in a 1/1000 carbendazim solution, and cultured in a 37 °C incubator (ZRG-250A-L cold light source artificial climate chamber, Shanghai Bailing Technology Co., Ltd., Shanghai, China) for 6 h. After being washed 2–3 times and submerged in clean water, they were then soaked in a 37 °C constant temperature and humidity box (BPS-250BC, Shanghai Yiheng Scientific Instrument Co., Ltd., Shanghai, China) for 40 h before being germinated in a 28 °C cultivation chamber (QHX-1000 artificial climate box, Wuhan Greenfafa institute of Novel Genechip R&D Co., Ltd., Wuhan, China) for 24 h. Seeds with good germination (using as standard the ratio of embryo shoot to root length is 1:2) were selected and placed in seedling bowls. These seedling bowls were placed in nursery trays and covered with vermiculite. The nursery trays were placed in the intelligent breeding factory (PGBS, Wuhan Greenfafa institute of Novel Genechip R&D Co., Ltd., Wuhan, China). The temperature was 32 °C under daylight and 26 °C in darkness. The humidity was maintained at a constant 60%. The photoperiod was set as a short-day condition (8 h of light and 16 h of dark) and a long-day condition (12 h of light and 12 h of dark). The rice breeding medium (60 L/bag) is specifically designed for rice breeding, from the Guangdong Shengsheng Agriculture Co., Ltd. (Guangzhou, China), and dry-wet alternate irrigation with tap water was used. Five days after sowing, 4 g of urea is applied to each plot on both sides of the rice leaves during the one-leaf-one-heart stage. Ten days later, 8 g of chemical fertilizer (nitrogen:phosphorus:potassium = 15:15:15) was applied. At the late stage of tillering and early stage of heading, 5 g of the 15:15:15 compound fertilizer containing nitrogen, phosphorus, and potassium was applied. Only yellow sticky boards were used to prevent pests throughout the cultivation process, without using any pesticides. The plant density is maintained at 15 seedling bowls (size: upper diameter 110 mm, lower diameter 750 mm, and height 90 mm) per nursery tray (size: 56 cm × 38 cm × 8 cm), with two rice seedlings per seedling bowl (Figure S1). At least 10 seedling trays were planted for each variety under long- or short-day conditions. Three independent biological replicates were performed.

### 2.2. Chemicals

Chromatographic grade methanol, acetonitrile (Thermo Fisher, Waltham, MA, USA), and acetic acid (Sigma-Aldrich, Saint Louis, MO, USA) were used for metabolic analysis. The internal standard for metabolic sample preparation is lidocaine (Sigma-Aldrich, Saint Louis, MO, USA). The standards were purchased from Aladdin Co., Ltd. (Shanghai, China). The standard solutions were stored at −80 °C. The substance was dissolved based on its solubility and stored at −20 °C. Ultra-pure water (deionized water) was prepared by the Barnstead pure water system (Thermo Scientific, Waltham, MA, USA).

### 2.3. Sample Preparation and Metabolite Extraction

The rice grains were harvested from 5 individual plants under different regimes of short-day (SD) or long-day (LD) conditions and dried in a 37 °C oven for 3 days. Subsequently, samples were powdered using a grinding machine operating at 30 HZ for 2 min (MM400, Retsch, GmbH, Haan, Germany). Subsequently, 0.08–0.1 g of the powdered sample was measured, and three replicates were set. With a ratio of 10 mL/g, the extraction liquid (70% methanol) was added and then vortexed and ultrasonicated for 10 min, with this process being repeated three times. After centrifugation at 12,000 rpm at 4 °C for 10 min (Eppendorf, 5430R, Hamburg, Germany), the supernatant (300 μL) was absorbed with a syringe. Then, the extracts were filtered through 0.22 μm microfilters prior and filtered into a brown injection bottle containing an insertion tube. The extraction process was conducted entirely in the dark.

### 2.4. UHPLC-MS/MS Analysis

A high-throughput detection and quantitative analysis of the metabolites were performed using the UHPLC-MS/MS system (SHIMADZU Nexera X2 system coupled with SCIEX QTrap 6500+, Shimadzu Corporation, Kyoto, Japan). The samples were separated on a Shim-pack GISS C18 column (1.9 μm, 2.1 × 100 mm). The mobile phase was (A) water (containing 0.04% acetic acid, *v*/*v*) and (B) methanol (containing 0.04% acetic acid, *v*/*v*). The elution gradient: 0 min, 5% B; 11 min, 100% B; 15 min, 100% B; 15.5 min, 5% and 20 min, 5% B. The flow rate was 0.4 mL/min, and the column temperature was 40 °C. The mass spectrometry was performed with the ESI model, and the parameters were as follows: an ion spray voltage of 5500 V (+) and 4500 V (−); an ion source gas I (GSI), gas II (GSII), and curtain gas (CUR) of 50, 60, and 35 psi, respectively; and a temperature of 500 °C. The scanning mode was acquired as multiple reaction monitoring (MRM) experiments with the collision gas set to medium. The total schedule MRM cycle time was set to 0.8 s, whereas the dwell time for each MRM transition was automatically adjusted according to the number of recurring events.

### 2.5. Identification and Quantification of Metabolites

The metabolites were qualitatively identified based on the mass spectrum information and retention times relative to standards and database. The precursor ion and product ion values, retention times, and fragmentation patterns were compared with standards where available. When the standards were absent, the metabolites were identified according to local self-build database or a series public online database (HMDB, Massbank, MoNA, and METLIN). Ultimately, the mass spectrum raw data from quantitative assays were processed by MultiQuant 3.0.3 software, and the peak area was integrated with the MQ4 integration algorithm.

### 2.6. Statistical Analysis

The data quality was assessed, and substances with low responsiveness and substances whose peak values did not conform to the reference RT were removed. Principal component analysis (PCA) was employed to evaluate the differences in rice grain metabolites under different photoperiods and the degree of variance among samples within each group of rice grains. PCA was performed using the OmicShare tools, a free online platform for data analysis (www.omicshare.com/tools, accessed on 26 January 2024) [41]. The circle represents a 95% confidence interval. Statistical functions were used for comparison in R, version 3.5.0 (www.r-project.org, accessed on 26 January 2024). VIP (variable importance in projection) values obtained from orthogonal partial least-squares discriminant analysis (OPLS-DA) were generated using the R package MetaboAnalystR, which served as one of the thresholds for screening differentially accumulated metabolites between groups [42]. Higher VIP values indicate a greater contribution of that substance to distinguishing between the two groups. The circle represents a 95% confidence interval. For the differential accumulation of metabolites in KY131, HHZ, and KSR under SD and LD, screening was performed by setting the variable importance in the projection (VIP) ≥ 1, |log_2_FC| ≥ 1, and the *p* value < 0.05. The Kyoto Encyclopedia of Genes and Genomes (KEGG) database is a primary resource for metabolic research, used for the annotation analysis, enrichment, and pathway mapping of metabolites [43]. TBtools (2024.1.11) was used to perform heat maps [44], whereby the relative content of each metabolite in the matrix is logarithmically transformed (using a base of 10) and followed by normalization of each column. The dendrogram was used for a clustering analysis of metabolites based on relative content, grouping metabolites with similar accumulation trends in relative content in seeds under different photoperiods.

## 3. Results

### 3.1. Metabolite Profiles of Rice Grains under Different Photoperiods

**Figure 1:**
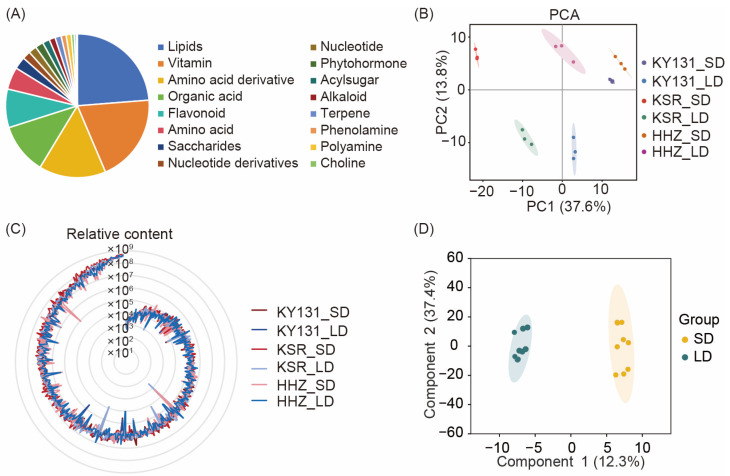
Metabolite determination based on rice grains harvested under different photoperiods (short-day, SD and long-day, LD). (**A**) 438 metabolites were identified in rice grains and classified into 16 categories based on the properties of the compounds. The measured metabolites are classified according to different categories and labeled with different colors, and their proportions are also listed. (**B**) Principal component analysis (PCA) of metabolites of three different varieties (KY131, HHZ, and KSR) under different photoperiods (SD and LD) showed good data repeatability. The circle represents a 95% confidence interval. (**C**) Overview of the relative metabolite content determination in rice grains under different photoperiods. The average values of metabolite content were utilized for ranking and a radar chart was used to show the relative metabolite content of different varieties under SD and LD conditions. The metabolite content under SD conditions are represented by red lines, while blue lines represent metabolites content under LD conditions. The higher the *Y*-axis value at which the outermost line is situated, the greater the level of metabolite content. The relative content of metabolites refers to the peak area of the metabolite peak graph. (**D**) Orthogonal partial least-squares discriminant analysis (OPLS-DA) of metabolites under different photoperiods (SD and LD). The circle represents a 95% confidence interval.

To detect the effects of light duration on the accumulation of nutrients in rice grains, we strictly planted rice under short-day (SD) and long-day (LD) conditions and performed a widely targeted metabolomic analysis method based on UHPLC-MS/MS to analyze the metabolomics of rice grains under different light cycle growth conditions. In total, 438 metabolites in rice grains were structurally detected and annotated, including vitamins and derivatives, lipids, flavonoids, organic acids, amino acids and derivatives, etc. (Figure 1A, Table S1). Through a principal component analysis (PCA) of the samples, the metabolic differences between groups and the degree of variability between samples in the same group were identified. The three repetitive samples were tightly clustered, suggesting high repeatability and reliability. The dispersion of PCA results of the metabolites in grains with different photoperiods also indicates the variability of rice grain metabolites under different photoperiods (Figure 1B). KSR and KY131 showed a clear distinction between the long and short photoperiods (corresponding to PC2), while the HHZ did not separate like others, indicating it is insensitive to the photoperiod. We performed a radar chart analysis to examine the differences in rice grain metabolites under different photoperiods. The relative metabolite content of each rice variety under different light treatments is connected into a line. The relative content of metabolites in rice under SD conditions, represented by the red lines, is primarily located in the outer circle. While under LD conditions, represented by the blue lines, the relative content of the metabolites in rice is mainly located in the inner circle, indicating that the accumulated relative metabolite content under SD conditions is significantly higher than that under LD conditions. The metabolic spectra of rice grains under different photoperiods show significant differences, which is consistent with the results of PCA (Figure 1C).

### 3.2. Overview of Different Categories of Metabolites in Rice Grains under Different Photoperiods

To characterize the differences in metabolite content caused by the photoperiods, we analyzed several important metabolite categories that determine the nutrition and quality. As a major metabolite category detected, most vitamins and derivatives showed a higher content in rice grains cultivated under SD conditions (Figure S2).

Free amino acids are essential to rice’s nutritional value and taste. Rice grains, excluding HHZ, accumulated more amino acids under short days, such as asparagine, glutamine, lysine, etc. Meanwhile, glutamic and aspartic acids accumulated more under LD conditions (Figure S3). The accumulation of free amino acids, alanine, glutamic acid, and aspartic acid, which enhance the sweetness of rice, varies among the three varieties under different photoperiods. The alanine content in KY131 and KSR is higher under SD conditions, while glutamate and aspartic acid are more abundant under LD conditions. However, its conversion products, such as glutamine, asparagine, and arginine, accumulate more under SD conditions.

Although the content is not as rich as carbohydrates and proteins, lipids in rice grains have a relatively substantial impact on the quality of rice. Therefore, we analyzed the accumulation of lipids in rice grains under different photoperiods. Most lipid contents accumulate more under SD conditions and less under LD conditions (Figure S4). The lipid content of KSR is notably higher than the other two varieties. In addition, the lipid structure of KSR seems to be different from KY131 and HHZ. However, lipids that accumulate less in KY131 and HHZ accumulate significantly in KSR. Comparatively, the lipid content in HHZ is relatively low, whether it is SD or LD.

Part of the flavonoid content accumulates more under SD conditions, while the opposite is in the other parts (Figure S5). The flavonoid accumulation patterns of KY131 and KSR exhibit similarities, whereas HHZ shows significant differences.

### 3.3. Differentially Accumulated Metabolites in Rice Grains under Different Photoperiods

**Figure 2:**
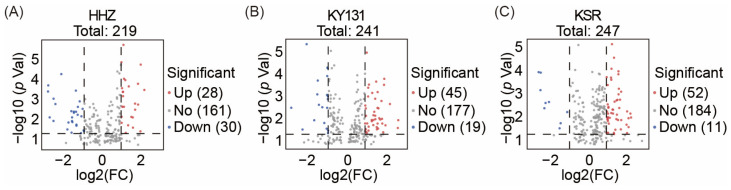
Up-regulated and down-regulated differentially accumulated metabolites (DAMs) in rice grains among three varieties under different photoperiods. (**A**) Volcano plot of DAMs in HHZ rice grains. (**B**) Volcano plot of DAMs in KY131 rice grains. (**C**) Volcano plot of DAMs in KSR rice grains. DAMs within three kinds of rice grains under varying photoperiods were screened using the criteria VIP ≥ 1, *p* value < 0.05, and absolute value of |log_2_FC| ≥ 1. The red circular dots represent up-regulated DAMs, the blue circular dots represent down-regulated DAMs, and the grey circular dots represent metabolites with insignificant differences.

To get insight into the metabolic differences and identify differentially accumulated metabolites (DAMs) under different photoperiods in three kinds of rice grains, an orthogonal partial least-squares discriminant analysis (OPLS-DA) was performed. The significant difference between the horizontal axis indicated the difference in rice grain metabolites between the two treatments (SD and LD). The vertical axis represents differences between samples within the group, showing slight differences in metabolites exhibited by different varieties due to their characteristics (Figure 1D). Each metabolite was assigned a variable importance in the projection (VIP) value through OPLS-DA, which represents the contribution of the substance to distinguishing between two groups. Therefore, we use VIP ≥ 1 as the screening condition to filter the metabolites first when selecting DAMs. Then, the criteria were a *p* value < 0.05 and |log_2_FC| ≥ 1 to filter out the significant DAMs within each rice grain under different photoperiods. According to the above screening rules, there were 58 DAMs (28 up-regulated and 30 down-regulated) found in HHZ (Figure 2A, Table S2), 64 DAMs (45 up-regulated and 19 down-regulated) were found in KY131 (Figure 2B, Table S3), and 63 DAMs (52 up-regulated and 11 down-regulated) were found in KSR (Figure 2C, Table S4). In KSR and KY131, exposure to SD conditions resulted in more up-regulated DAMs. While, in HHZ, there was little difference in the number of up-regulated and down-regulated metabolites caused by the photoperiods. In summary, different photoperiods cause differences in metabolite accumulation, and SD is a more influential condition for metabolite accumulation.

### 3.4. KEGG Pathway Annotation of the Differentially Accumulated Metabolites

**Figure 3:**
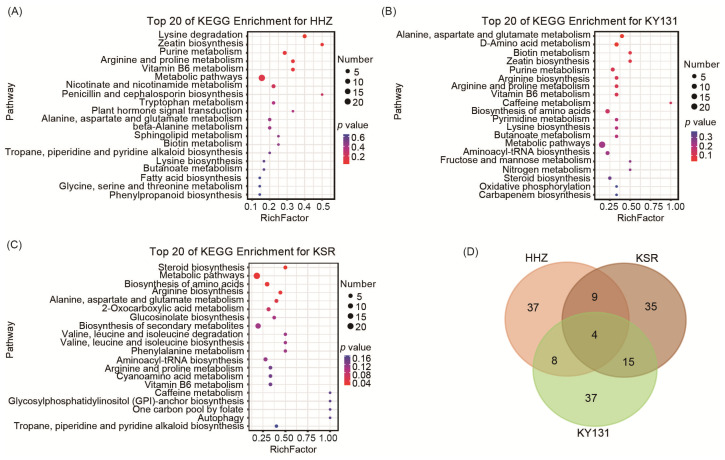
Differential metabolite analysis of HHZ, KY131, and KSR under different photoperiods. (**A**) The Kyoto Encyclopedia of Genes and Genomes (KEGG) analysis of DAMs under LD and SD conditions for HHZ. (**B**) KEGG analysis of DAMs under LD and SD conditions for KY131. (**C**) KEGG analysis of DAMs under LD and SD conditions for KSR. The size of the circle represents the quantity of enriched metabolites, with a deeper red color indicating higher significance. (**D**) Veen plot reveals the intersection and differences of DAMs among three varieties under different photoperiods. The overlapping portions indicate the number of differential metabolites identified among two or three varieties.

To analyze the enrichment of these DAMs, we conducted a Kyoto Encyclopedia of Genes and Genomes (KEGG) analysis. The KEGG pathway enrichment analysis indicated that the DAMs in HHZ under different photoperiods were prominently enriched in amino acid metabolisms, vitamin B3 and B6 metabolisms, and plant hormones, such as zeatin and brassinosteroids, etc. (Figure 3A). In addition to the enrichment of various amino acids and vitamin B6, the differential metabolites of the rice grains harvested by KY131 under different photoperiods were enriched in the sulfur relay system, fructose and mannose metabolisms, ABC transporters, taurine and hydrogen metabolisms, and steroid biosynthesis (Figure 3B). The metabolites that differ in KSR under different photoperiods were also enriched in various amino acid metabolisms and the vitamin B6 metabolism (Figure 3C). In conclusion, the photoperiod influences the accumulation of amino acids and vitamin B6 in rice grains.

### 3.5. Key Significantly Differentially Accumulated Metabolites

**Figure 4:**
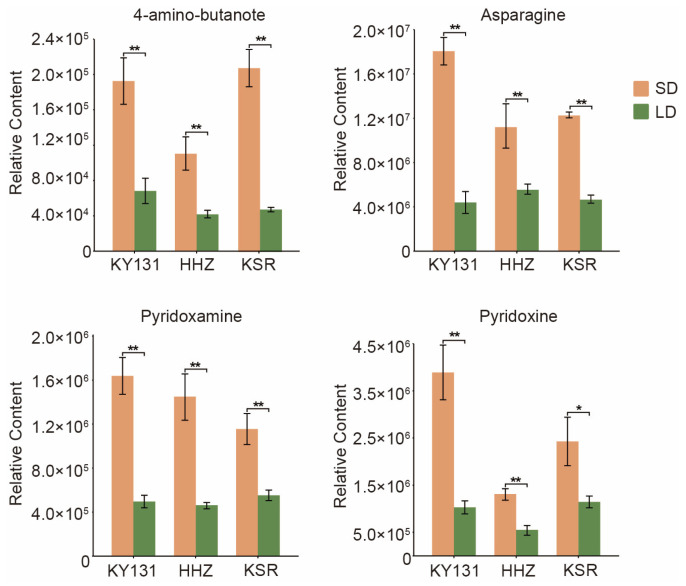
The relative content of key DAMs under different photoperiods in three varieties. The orange column represents the relative content of metabolites under SD conditions, and the green column represents the relative content of metabolites under LD conditions. *p* values are from the *t*-test. * represents *p* < 0.05 and ** represents *p* < 0.01.

A Venn diagram analysis was performed to identify the key DAMs affected by the photoperiod. The result revealed that four metabolites overlapped among the three pairwise comparisons (Figure 3D). These key DAMs include amino acids and derivatives, vitamins, and organic acids. The DAMs from the Venn diagram intersection contain two kinds of vitamin B6: pyridoxamine and pyridoxine. The results indicate that vitamin B6 accumulates more in rice grains under the SD condition. Asparagine, as an umami substance, was significantly more detected in rice grains under SD conditions than LD conditions. Rice grains under SD conditions produced more 4-Aminobutyric acid, which is often added to food or feed to improve health and promote growth due to its sedative effect (Figure 4). In conclusion, we identified more delicious and nutritious metabolites accumulated in rice grains under SD conditions, which may suggest that planting under SD conditions can be one of the ways to improve rice quality.

### 3.6. Differential Accumulation of Metabolites in the Vitamin B6 Pathway

**Figure 5:**
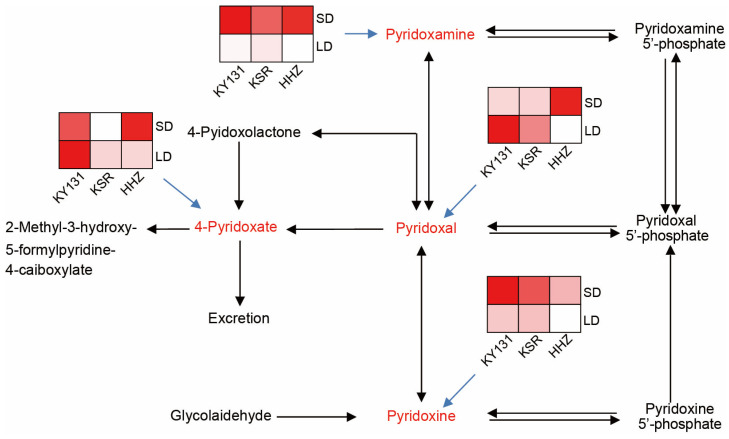
Key metabolites in the vitamin B6 pathway. Key metabolites are marked in red. The blue arrow signifies a heatmap representation of the key metabolites. The higher the metabolite content, the deeper red in the square of the heat map.

The enriched metabolites are mainly concentrated in the vitamin B6 pathway among three rice varieties under different photoperiods. Therefore, we mapped the metabolic pathways of vitamin B6 and identified the accumulation changes of related substances under different photoperiods. Vitamin B6, also known as pyridoxine, includes pyridoxine, pyridoxal, and pyridoxamine. The accumulation of pyridoxine, an active substance of Vitamin B6, is significantly higher in rice grains harvested under SD conditions than those under LD conditions. Correspondingly, the pyridoxal and 4-pyridoxate, which can convert to pyridoxal, accumulate more under LD conditions. In addition, pyridoxamine was potentially converted to pyridoxal, and its pattern was consistent with that of pyridoxine (Figure 5).

## 4. Discussion

Rice is a fundamental source of sustenance for half of the global populace, serving as a cornerstone for energy and nourishment [45]. Its widespread consumption implies enhancing nutritional value to address global nutritional needs [46]. Although strategies such as genetic hybrid breeding, molecular design breeding, and gene-edited breeding have great potential to improve rice nutrition, the time cost of these strategies is often high [47]. Recently, the rapid breeding strategy of changing the light source and adjusting the photoperiod to harvest seeds earlier has been more broadly applied [48]. However, it needs to be clarified how the photoperiod affects the accumulation of nutrient metabolites in rice grains, presenting a critical scientific inquiry concerning the imperative need to safeguard food security. With the ongoing advancement of plant factories, the precise cultivation of crops has emerged as a trend, which makes it possible to regulate field crops under controllable light and temperature conditions. In this study, three rice varieties were cultivated in a plant factory to investigate the impact of photoperiods on rice metabolomics. The results indicated that most metabolites are more likely to accumulate under short-day conditions, especially vitamin B6 and amino acids, with only a tiny portion accumulating less. Thus, SD cultivation in plant factories not only conserves energy costs but also enhances the nutritional value and sensory qualities of rice, making it an excellent and sustainable cultivation method.

Integrating advancements in light-emitting diodes (LEDs) with techniques like photoperiod manipulation holds promise for expanding the scope of rapid breeding methodologies [49,50]. However, it is noteworthy that despite the potential for adjusting photoperiods to expedite plant growth cycles there needs to be empirical validation regarding its nutritional advantages. In previous studies, the artificial manipulation of photoperiods in medicinal plant cultivation has significantly enhanced the core metabolite content. By analyzing the metabolic profiles of rice grains under SD and LD conditions, it has been observed that the accumulation of diverse metabolites experience a notable increase under short-day conditions (Table S1, Figures S2–S5). Vitamin B6, which includes pyridoxine, pyridoxal, and pyridoxamine, plays essential roles as a cofactor in a range of biochemical reactions, and promotes plant tolerance to high light and UV-B, hinting at a light-mediated regulation of vitamin B6 accumulation within the plant [51,52,53]. Notably, vitamin B6 is easily damaged by light or alkali and is not resistant to high temperatures [54]. The instability of vitamin B6 when exposed to light also partly explains the low levels of active vitamin B6 (pyridoxine) in rice grains under LD conditions. Therefore, we speculate that the changes in the photoperiod are beneficial for improving the nutritional composition of rice.

Amino acid metabolism exhibits a strong association with light spectrum exposure. The taste of rice is partly dependent on its amino acid content [55]. For instance, the composition of glutamine, asparagine, serine, and glycine in lettuce leaves is influenced by light spectra. Green light enhances the amino acid content, leading to higher levels of glutamic acid, asparagine, and tyrosine in green lettuce leaves. In contrast, red light increases the content of glutamic acid, asparagine, aspartic acid, serine, glutamine, threonine, and arginine in leaves [56]. However, prior research has yet to fully elucidate the impact of the photoperiod on amino acid accumulation in plants. In this study, enriched DAMs were in amino acid synthesis pathways (Figure 3A–C). Asparagine in rice grains significantly increased under SD conditions compared to LD conditions (Figure 4). In addition, the essential amino acids, phenylalanine, methionine, lysine, threonine, tryptophan, leucine, isoleucine, and valine accumulated more under SD conditions (Figure S3). This finding highlights the significance of understanding the impact of photoperiods on amino acid synthesis in rice production.

The plant metabolome exhibits a wide diversity. To determine the impact of differences in photoperiod on the metabolite content of different varieties of rice grains and eliminate the differences in the response of rice varieties to photoperiod due to their characteristics, we selected the rice variety KY131 cultivated in the north, the rice variety HHZ grown in the south, and a special variety of the Dong nationality in China named KSR, as representatives. Exposure to different photoperiods typically results in increased levels of metabolites in rice grains grown under SD conditions. There is good reproducibility in KY131 and KSR. However, some metabolites in HHZ showed an opposite trend, indicating varietal specificity. KSR is a variety with high nutritional content unique to the Dong nationality [40]. In our metabolite profiling, the content of amino acids and lipids in KSR is significantly higher than the other two varieties, which confirms that our results are consistent with the characteristics of the variety itself, indicating its authenticity and credibility. Furthermore, it is noteworthy that planting KSR under SD conditions can further enhance its amino acid content. This further proves that SD conditions are beneficial for improving the nutritional content of rice grains.

## 5. Conclusions

This study demonstrated that a difference in photoperiod leads to a different accumulation of metabolites in rice grains. Planting under a short-day condition significantly increases the content of numerous metabolites, including amino acids, vitamins, and flavonoids. This indicates that planting under short days helps improve rice nutrition and taste quality. These findings provide insights into regulating plant growth and development and nutrient reprogramming by photoperiod, thus opening avenues for cultivating rice varieties with a higher nutrient content.

## Data Availability

The original contributions presented in the study are included in the article/Supplementary Materials, further inquiries can be directed to the corresponding authors.

## Supplementary Materials

The following supporting information can be downloaded at: https://www.mdpi.com/article/10.3390/foods13101544/s1, Figure S1. The procedure for cultivating rice in PGBS and QHX-1000 involves the following steps: washing the seeds, soaking, germination, sowing, irrigation, and fertilizer application; Figure S2: Heat map of vitamins and their derivatives content; Figure S3: Heat map of amino acid content; Figure S4: Heat map of lipid content; Figure S5: Heat map of flavonoid content. The software TBtools is used for heatmaps, whereby the relative content of each metabolite in the matrix is logarithmically transformed (using a base of 10) and followed by normalization of each column. The colors in the heatmap reflect the levels of relative metabolite content, where red indicates a higher relative content of metabolites and blue indicates a lower relative content of metabolites. The dendrogram was used for a clustering analysis of the metabolites based on relative content, grouping together metabolites with similar accumulation trends in relative content in seeds under different photoperiods; Figure S6: Characterization of VB6 by UHPLC-MS. (A) XIC (extracted ion chromatogram) of compound pyridoxine; (B) XIC of compound pyridoxamine; Table S1: Relative content of metabolites in rice grains under SD and LD conditions; Table S2: Significant differentially accumulated metabolites between rice grains under SD and LD conditions in HHZ; Table S3: Significant differentially accumulated metabolites between rice grains under SD and LD conditions in KY131; Table S4: Significant differentially accumulated metabolites between rice grains under SD and LD conditions in KSR. Screening was performed in setting the variable importance in projection as VIP ≥ 1, |log_2_FC| ≥ 1, and a *p* value < 0.05.
