# Supplementary material for: Photoperiod-Dependent Nutrient Accumulation in Rice Cultivated in Plant Factories: A Comparative Metabolomic Analysis
Source: Foods. 2024 May 16;13(10):1544. doi: 10.3390/foods13101544 (PMC11121446; doi:10.3390/foods13101544)
Supplement: Supplementary file 1 [file foods-13-01544-s001.zip › Supplementary Figures.pdf]

foods

Supplementary Figures for

Photoperiod-dependent Nutrient Accumulation in Rice Cultivated in Plant Factories: A Comparative Metabolomic Analysis

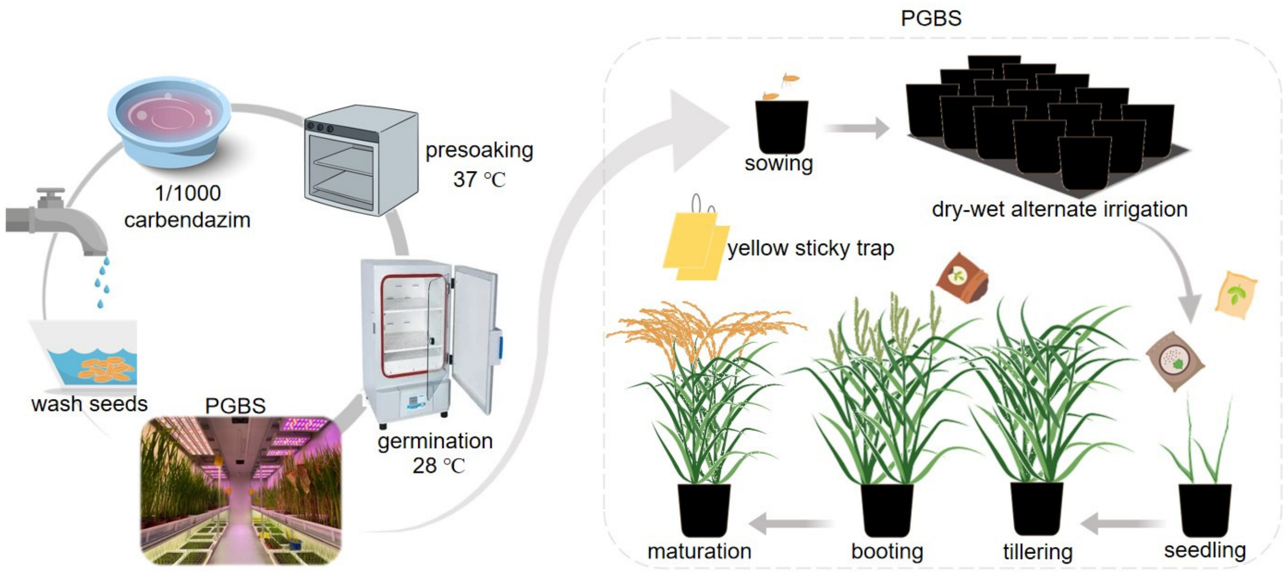

Figure S1 The procedure for cultivating rice in PGBS and QHX-1000 involves the following steps: washing the seeds, soaking, germination, sowing, irrigation and fertilizer application.

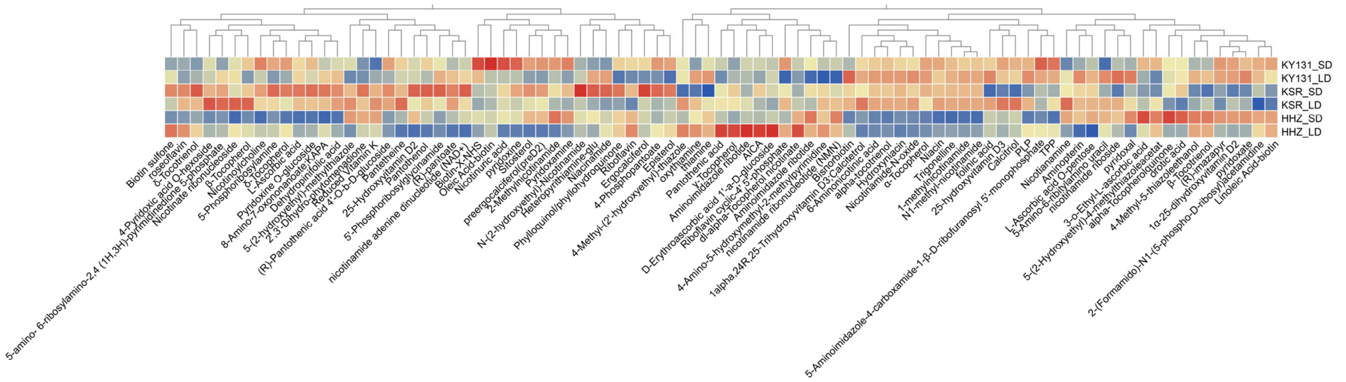

Figure S2 Heat map of vitamins and their derivatives content. The software TBtools is used for heatmaps, whereby the relative content of each metabolite in the matrix is logarithmically transformed (using a base of 10) and followed by normalization of each column. The colors in the heatmap reflect the levels of relative metabolite content, where red indicates higher relative content of metabolites and blue indicates lower relative content of metabolites. The dendrogram was used for clustering analysis of metabolites based on relative content, grouping together metabolites with similar accumulation trends of relative content in seeds under different photoperiods.

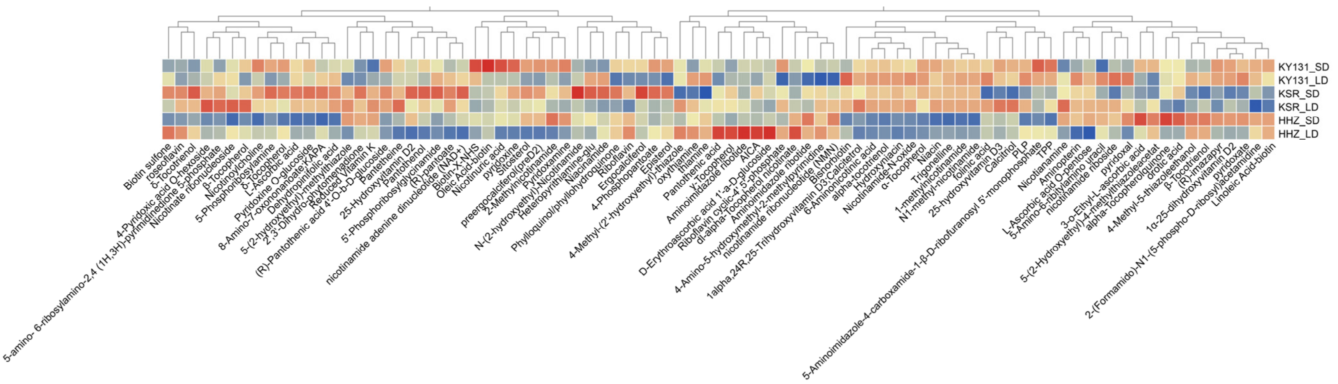

Figure S3 Heat map of amino acid content. The software TBtools is used for heatmaps, whereby the relative content of each metabolite in the matrix is logarithmically transformed (using a base of 10) and followed by normalization of each column. The colors in the heatmap reflect the levels of relative metabolite content, where red indicates higher relative content of metabolites and blue indicates lower relative content of metabolites. The dendrogram was used for clustering analysis of metabolites based on relative content, grouping together metabolites with similar accumulation trends of relative content in seeds under different photoperiods.

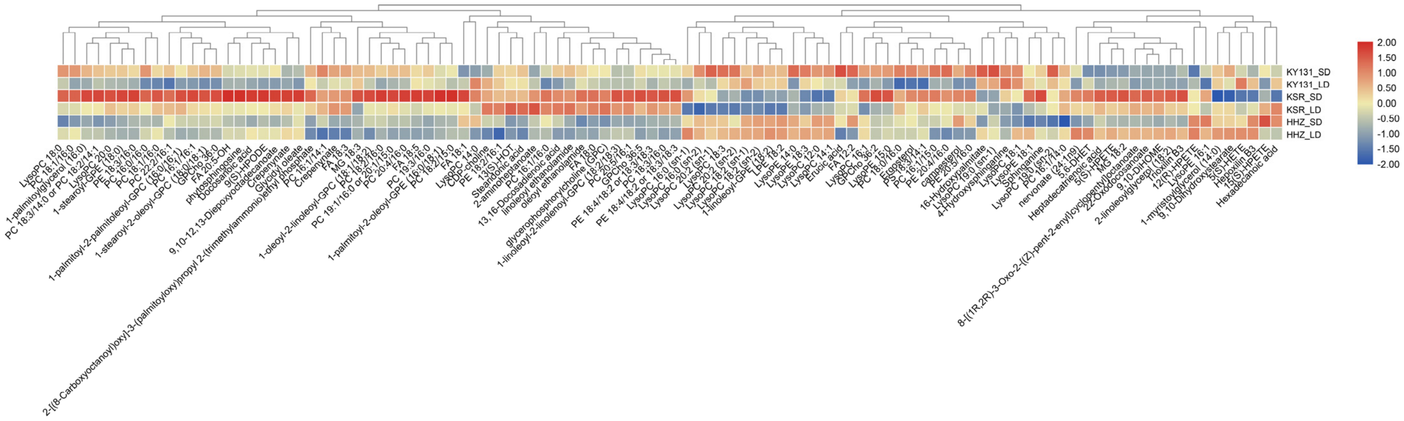

Figure S4 Heat map of lipid content. The software TBtools is used for heatmaps, whereby the relative content of each metabolite in the matrix is logarithmically transformed (using a base of 10) and followed by normalization of each column. The colors in the heatmap reflect the levels of relative metabolite content, where red indicates higher relative content of metabolites and blue indicates lower relative content of metabolites. The dendrogram was used for clustering analysis of metabolites based on relative content, grouping together metabolites with similar accumulation trends of relative content in seeds under different photoperiods.

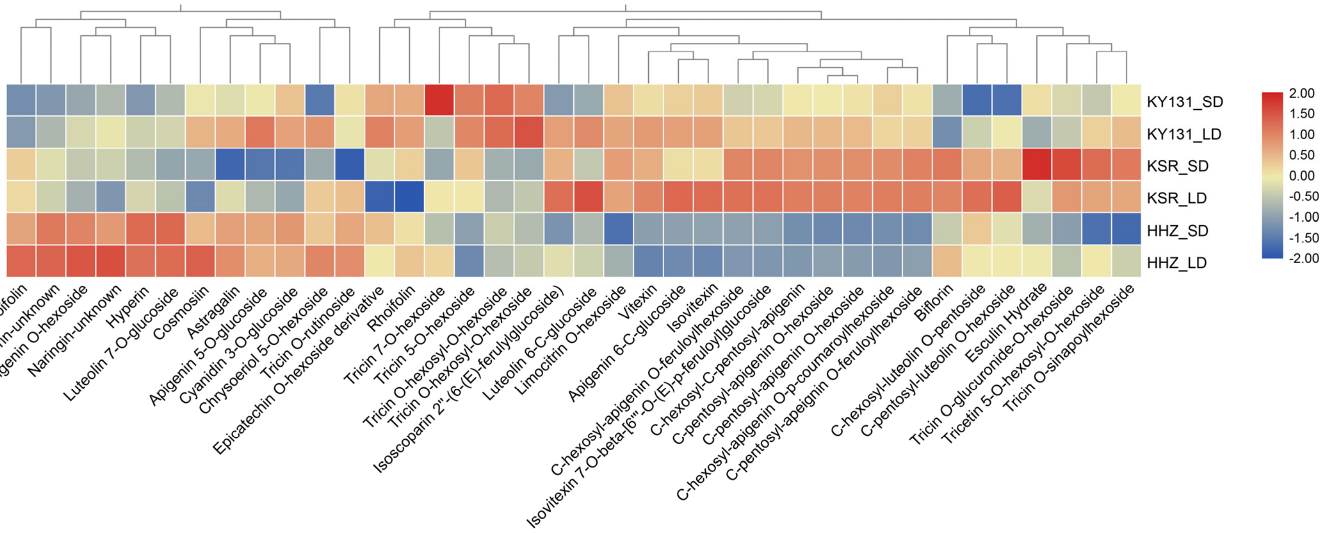

Figure S5: Heat map of flavonoid content. The software TBtools is used for heatmaps, whereby the relative content of each metabolite in the matrix is logarithmically transformed (using a base of 10) and followed by normalization of each column. The colors in the heatmap reflect the levels of relative metabolite content, where red indicates higher relative content of metabolites and blue indicates lower relative content of metabolites. The dendrogram was used for clustering analysis of metabolites based on relative content, grouping together metabolites with similar accumulation trends of relative content in seeds under different photoperiods.

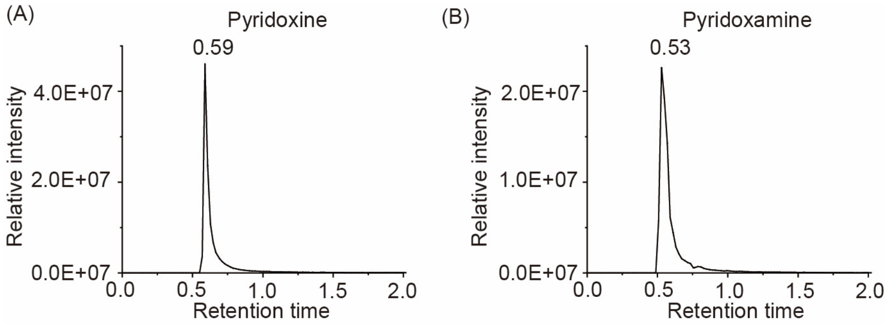

Figure S6 Characterization of VB6 by UHPLC-MS. (A) XIC (extracted ion chromatogram) of compound pyridoxine; (B) XIC of compound pyridoxamine.
